# Voids and compositional inhomogeneities in Cu(In,Ga)Se_2_ thin films: evolution during growth and impact on solar cell performance

**DOI:** 10.1080/14686996.2018.1536679

**Published:** 2018-11-19

**Authors:** Enrico Avancini, Debora Keller, Romain Carron, Yadira Arroyo-Rojas Dasilva, Rolf Erni, Agnieszka Priebe, Simone Di Napoli, Martina Carrisi, Giovanna Sozzi, Roberto Menozzi, Fan Fu, Stephan Buecheler, Ayodhya N. Tiwari

**Affiliations:** a Laboratory for Thin Films and Photovoltaics, Empa-Swiss Federal Laboratories for Materials Science and Technology, Duebendorf, Switzerland; b Electron Microscopy Center, Empa-Swiss Federal Laboratories for Materials Science and Technology, Duebendorf, Switzerland; c Laboratory for Mechanics of Materials and Nanostructures, Empa- Swiss Federal Laboratories for Materials Science and Technology, Thun, Switzerland; d Department of Engineering and Architecture, University of Parma, Parma, Italy

**Keywords:** Cu(In, Ga)2, multistage coevaporation, STEM/EDX, 50 Energy Materials, 209 Solar cell / Photovoltaics, 306 Thin film / Coatings; 503 TEM, STEM, SEM, 302 Crystallization / Heat treatment / Crystal growth

## Abstract

Structural defects such as voids and compositional inhomogeneities may affect the performance of Cu(In,Ga)Se_2_ (CIGS) solar cells. We analyzed the morphology and elemental distributions in co-evaporated CIGS thin films at the different stages of the CIGS growth by energy-dispersive x-ray spectroscopy in a transmission electron microscope. Accumulation of Cu-Se phases was found at crevices and at grain boundaries after the Cu-rich intermediate stage of the CIGS deposition sequence. It was found, that voids are caused by Cu out-diffusion from crevices and GBs during the final deposition stage. The Cu inhomogeneities lead to non-uniform diffusivities of In and Ga, resulting in lateral inhomogeneities of the In and Ga distribution. Two and three-dimensional simulations were used to investigate the impact of the inhomogeneities and voids on the solar cell performance. A significant impact of voids was found, indicating that the unpassivated voids reduce the open-circuit voltage and fill factor due to the introduction of free surfaces with high recombination velocities close to the CIGS/CdS junction. We thus suggest that voids, and possibly inhomogeneities, limit the efficiency of solar cells based on three-stage co-evaporated CIGS thin films. Passivation of the voids’ internal surface may reduce their detrimental effects.

## Graphical Abstract

**Figure:**
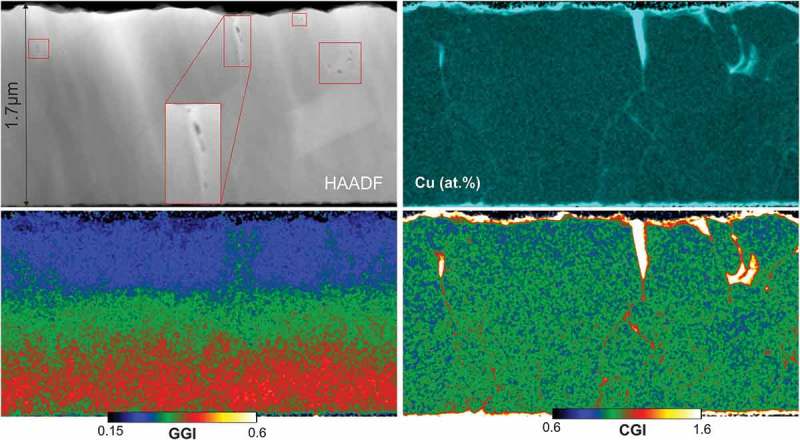

## 1. Introduction

Recent improvements in the development of Cu(In,Ga)Se_2_ (CIGS) solar cells have mostly focused on the optimization of the charge-selective contacts and on the addition of alkali metals for interface modification and doping [1]. Further increases of the device efficiency might be prevented by the presence of structural defects in the CIGS layer, such as voids and compositional inhomogeneities. A full understanding is desired on how these structural defects are created during multistage coevaporation of CIGS layers, and on how they may affect the solar cell performance.

The CIGS bandgap depends on the [Ga]/([Ga]+[In]) (GGI) ratio [2] ranging from 1.0 eV for pure CuInSe_2_ to 1.7 eV for pure CuGaSe_2_. Inhomogeneous lateral distributions of the group-III elements In and Ga may therefore lead to bandgap fluctuations. Bandgap fluctuations in CIGS thin films have been investigated due to their potential impact on the photovoltaic performance of solar cells [3]. Especially, bandgap fluctuations may limit the open-circuit voltage (V_oc_) [4,5]. Compositional and bandgap fluctuations on a microscopic scale have been investigated by different laterally resolved techniques [3,6–10]. Abou-Ras et al. [3] measured inhomogeneities in the Ga and In lateral distribution by scanning transmission electron microscopy combined with energy-dispersive X-ray spectroscopy (STEM/EDX), although predicting negligible impact on the solar cell performance. Other authors suggested a larger impact of compositional inhomogeneities on the V_oc_ [5].

Another structural defect in CIGS films could be the presence of voids. Voids can be defined as volumes of missing material within a thin film. The presence of voids might affect the device performance by the introduction of unpassivated, highly recombinative free surfaces close to the charge selective contact. No specific literature on that topic is available so far for CIGS solar cells.

A good understanding has been achieved regarding the formation of beneficial GGI gradings across the depth of the CIGS absorbers [11,12], and also the existence of voids in the CIGS layer has previously been reported [13,14]. However, the formation mechanism of lateral inhomogeneities of the CIGS composition has not been investigated yet.

Several factors of the CIGS deposition method may affect the formation of voids and inhomogeneities. For example, high substrate temperatures may enhance elemental diffusion, whereas the presence of alkali elements during co-evaporation of CIGS thin films may reduce elemental diffusivity and grain growth especially at low deposition temperatures (< 450 °C) [15].

Here, we report the formation of voids and inhomogeneous elemental distributions in low-temperature multi-stage co-evaporated CIGS absorbers with no presence of alkali elements during growth. The elemental distribution and film texture were analyzed at different stages of deposition sequence, which allows us to propose a model for the formation mechanism of voids and compositional inhomogeneities. We studied the impact of such structural defects on the photovoltaic performance by two-dimensional and three-dimensional device simulations.

## 2. Experimental details

CIGS layers were deposited by multistage evaporation in an in-house built unit on soda-lime glass substrate, as described elsewhere [11]. A silicon oxide barrier layer was deposited between the glass and the sputtered molybdenum back contact in order to prevent diffusion of alkali elements into the CIGS.

It is important to note that the measurement analyses were performed on CIGS films that yield high efficiency solar cells. The best solar cell grown from the completed CIGS film analyzed here had a V_oc_ of 725 mV, a fill factor of 77.3%, a short-circuit current density (J_sc_) of 34.8 mA/cm^2^ and a power conversion efficiency of 19.5% (with a 105 nm-thick MgF_2_ anti-reflective coating).

Multi-stage co-evaporation processes for CIGS deposition consist of the initial evaporation of (In,Ga)_2_Se_3_, subsequent addition of Cu until a Cu-rich CIGS film is obtained (first stoichiometry point), and a final evaporation of (In,Ga)_2_Se_3_ in order to make the overall composition Cu-poor. The Cu-rich stage coincides with the surface segregation of Cu-Se phases and to a re-crystallization of the CIGS film to form bigger grains with reduced defect density [16]. After the first stoichiometry point, Cu is added in excess with a relative increase in the Cu concentration of 5%–10% relative to stoichiometric concentrations. The first stoichiometry point is identified by a sudden increase in power needed to maintain constant temperature of the substrate, due to the difference in thermal emissivity of segregating Cu-Se phases at the CIGS surface (defined as end-point detection [17,18]).

Three samples were extracted at different stages of the deposition: Sample 1 just before the first stoichiometry point. Sample 2 was extracted just after the first stoichiometry point, without additional Cu excess. Sample 1 and Sample 2 were produced on the same substrate and during the same deposition process, by intentionally exploiting a known feature of our deposition equipment to achieve a gradient of the Cu evaporation rate at the chosen substrate position, and by carefully selecting the time of interruption of the Cu evaporation rate and subsequent sample extraction. Sample 3 corresponds to a full solar cell stack including a complete CIGS deposition, NaF and RbF postdeposition treatment (PDT), CdS chemical bath deposition, sputtered unintentionally doped ZnO and ZnO:Al (2 wt.% Al_2_O_3_) window layers, Ni/Al grids, and MgF_2_ antireflective coating (ARC). All processes are described in detail in a previous publication [11].

The PDT and the deposition of CdS and window layers are not expected to affect the GGI grading and lateral elemental distribution on a micrometer scale. PDT is in fact performed at a temperature approximately 100 °C below the deposition temperature, and the presence of alkali elements is known to further reduce group-III element diffusivities [19]. PDTs were reported to modify the surface and GB chemical composition at the surface [20] and grain boundaries [21] only in regions limited to a few nanometers. Therefore, it is justified to assume any micrometer-scale GGI inhomogeneities are present already at the end of the CIGS deposition before the PDT.

The average composition of the CIGS films was measured by X-ray fluorescence (XRF), previously calibrated by standards with known composition in an in-house built measurement unit. Details can be found in previous publications (standard calibration in the publication by Carron et al. [22]).

Specimens for TEM were prepared on lifted-off CIGS films on Si wafer substrates by conventional mechanical polishing and Ar^+^-ion milling with liquid N_2_ cooling as described previously by Keller et al. [21]. A final specimen thickness between 50 nm and 100 nm was obtained.

High angle annular dark-field (HAADF) STEM micrographs were obtained using a Titan Themis TEM/STEM (FEI, Hillsboro, OR, USA) operated at 300 kV with a 3.9 nA beam current. EDX mapping was performed with a lateral sampling from 1.16 nm to 4.5 nm using a SuperX EDX detector in the same experimental setup. The EDX spectra were analyzed using the softwares Velox (FEI, Hillsboro, OR, USA) and DigitalMicrograph (Gatan, Pleasanton, CA, USA). Atomic composition (at.%) maps were calculated by the software Velox using the following specifications: multi-polynomial background correction, parabolic background order, 100 nm thickness, 5.7 g/cm^3^ density, Schreiber-Wims model for the ionization cross section. Quantification was performed on the Cu, In, Ga, Se, Na, Rb, O signals, with additional deconvolutions of the K, Mo, Zn, Cd, S and Si signals. We calculated GGI and CGI maps based on the Cu, In and Ga at.% maps. The average Cu, In and Ga concentrations were re-calibrated so that the average CGI and GGI of the calculated map would match the average values previously determined by XRF. This was necessary due to an over-estimation of the In signal in the quantification of STEM-EDX at.% maps. We consider the XRF values more reliable than the STEM/EDX quantification, after a careful calibration of the XRF-based quantification method by inductively coupled plasma optical emission spectrometry (ICP-OES), as described in an earlier publication [22].

**Figure 1.**
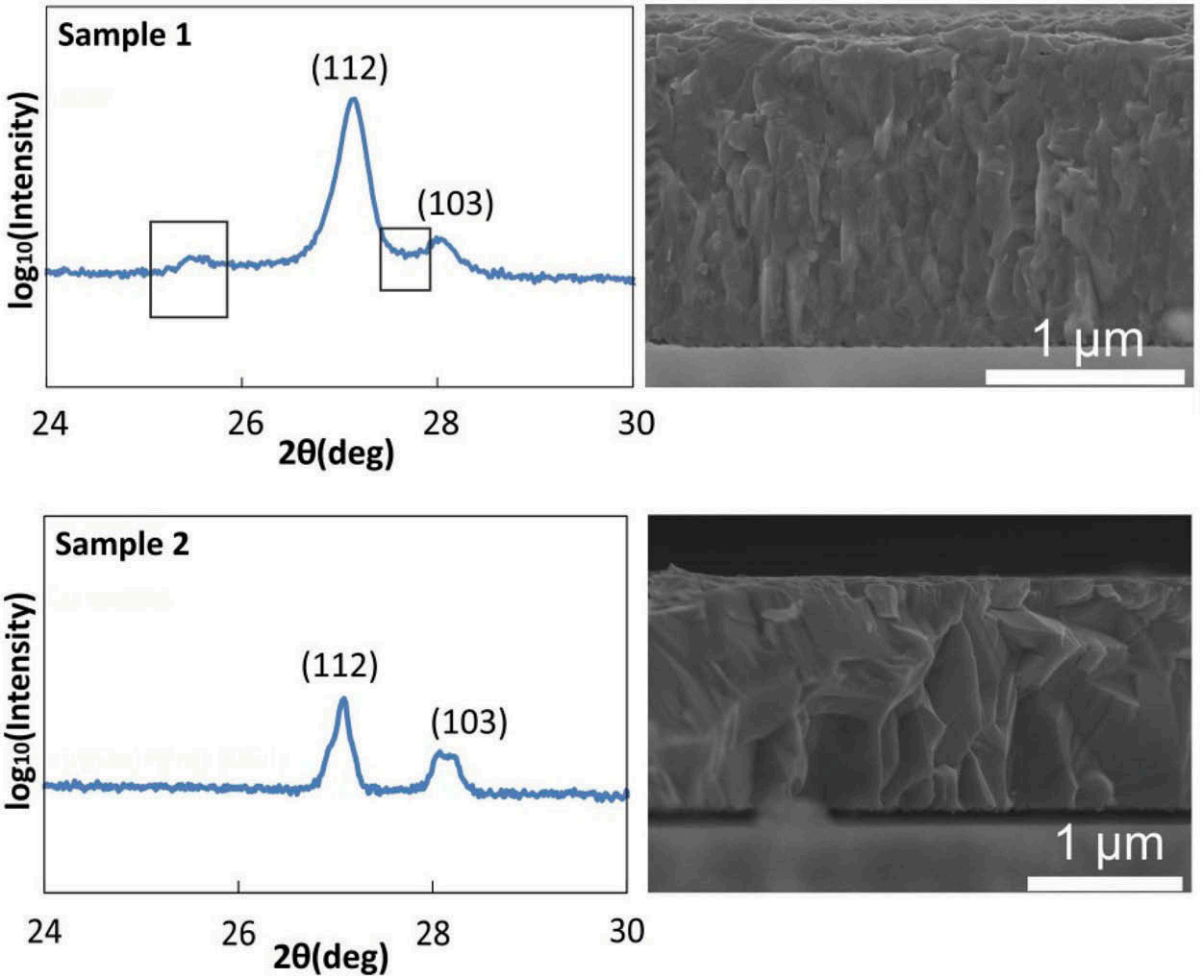
Selected sections of XRD patterns of Samples 1 (growth interrupted before the 1^st^ stoichiometry point) and 2 (growth interrupted just after the 1^st^ stoichiometry point) with corresponding cross-sectional SEM micrographs.

From Sample 1, a slightly thinner TEM specimen was obtained as compared to those obtained for Sample 2 and Sample 3. In combination with the choice of a high resolution (1.16 nm/pixel), this resulted in CGI and GGI maps with a low signal to noise ratio (SNR). In order to make the data more readable and comparable, the In, Cu and Ga at.% maps were binned two times before calculating the CGI and GGI maps (bilinear binning of neighboring pixel, DigitalMicrograph software). Further smoothening (DigitalMicrograph software) was applied to the already-calculated CGI and GGI maps. The binning and smoothening were used exclusively for the CGI and GGI maps of Sample 1. This did not lead to any loss of information which may affect the interpretation. A comparison is shown in the supporting information (Figure 1 supp.) between the CGI and GGI maps calculated from the binned and the nonbinned Cu, In, and Ga maps. A comparison between the non-smoothened and the smoothened GGI and CGI maps is also shown. Both comparisons indicate that the binning and the smoothening only improve the SNR without interfering with the interpretation for our purposes. The same modified analysis was needed also for one CGI and GGI map of Sample 2 in the supporting information (specified in the caption).

In some other cases (specified in the text) a Jeol 2200FS TEM/STEM microscope was used, operated at 200 kV. EDX spectra were analyzed by using the software DigitalMicrograph (Gatan, Pleasanton, CA, USA). The Cd and In signals were separated by multiple linear least squares (MLLS) fitting the measured spectra to previously measured Cd and In reference spectra.

X-ray diffraction was measured in a Bragg-Brentano configuration from 10 to 60° (2θ) and 0.0167° step intervals with a X’Pert PRO θ-2θ (PANanalytical, Almelo, Netherlands) scan using Cu-Kα1 radiation.

Scanning electron microscopy (SEM) cross-sectional micrographs were obtained using a Hitachi S-4800 (Tokyo, Japan) unit with electron acceleration voltage of 5 kV and a working distance of 4 mm. The sample cross section was prepared by cleaving the sample and substrate stack just before the measurement.

Compositional depth profiles were measured by secondary ion mass spectroscopy (SIMS) with a time-of flight (TOF) mass spectrometer, using a TOF-SIMS [5] by ION-TOF (Muenster, Germany). Bi was used as primary ion gun with 25 kV acceleration voltage and 1 pA current, whereas O_2_ was used for sputtering with 2 kV acceleration voltage and 400 nA current. The measurement area was 100 × 100 µm^2^ and the sputtering area was 300 × 300 µm^2^.

A Focused Ion Beam Scanning Electron Microscopy (FIB-SEM) Lyra3 by Tescan (Brno, Czech Republic) was used to etch the surface of a sample. The sample surface was sputtered with 20 keV energy Ga+ beam at 182 pA ion current. SEM images were acquired in-situ using 15 keV electron beam.

## 3. Results

### 3.1. Film characterization

Figure 1 shows a selected section of X-ray diffraction (XRD) patterns and SEM cross section images of Sample 1 and Sample 2. All measurements were performed *ex situ* at room temperature. SEM cross sections indicate that larger, well defined grains are formed when the CIGS film turns from Cu-poor (Sample 1) to Cu-rich (Sample 2). This is consistent with the recrystallization process typically observed after the first stoichiometry point [23]. The grain size and texture of Sample 2 are similar to those typically observed for completed CIGS growths (e.g. in reference [24]). Two prominent XRD peaks are observed for both samples at ~ 27° and ~ 28°, corresponding to the expected positions of the (112) and (103) reflections of the CIGS alpha-phase. The XRD pattern of Sample 1 exhibits a further small peak at ~ 25° and a large shoulder between the 112 and 103 peaks. These two features have been described as the signature for a large density of stacking faults in CIGS films that did not undergo a Cu-rich stage [25]. These features are not visible in Sample 2. This is consistent with the re-crystallization of the CIGS film and annihilation of stacking faults at the Cu-poor to Cu-rich transition in low-T multi-stage deposition with no alkali elements [15,23,25].

XRF measurements indicate that both samples have nearly stoichiometric or stoichiometric Cu amounts (CGI 0.98 ± 0.02 for Sample 1 and 1.00 ± 0.02 for Sample 2). It is not surprising that Sample 2 exhibits Cu-Se phase segregation despite the stoichiometric Cu concentration. Phase diagrams indicate in fact that Cu-Se phases may segregate already at CGIs above 0.95 [26].

### 3.2. Evolution of the compositional distribution and grading

**Figure 2.**
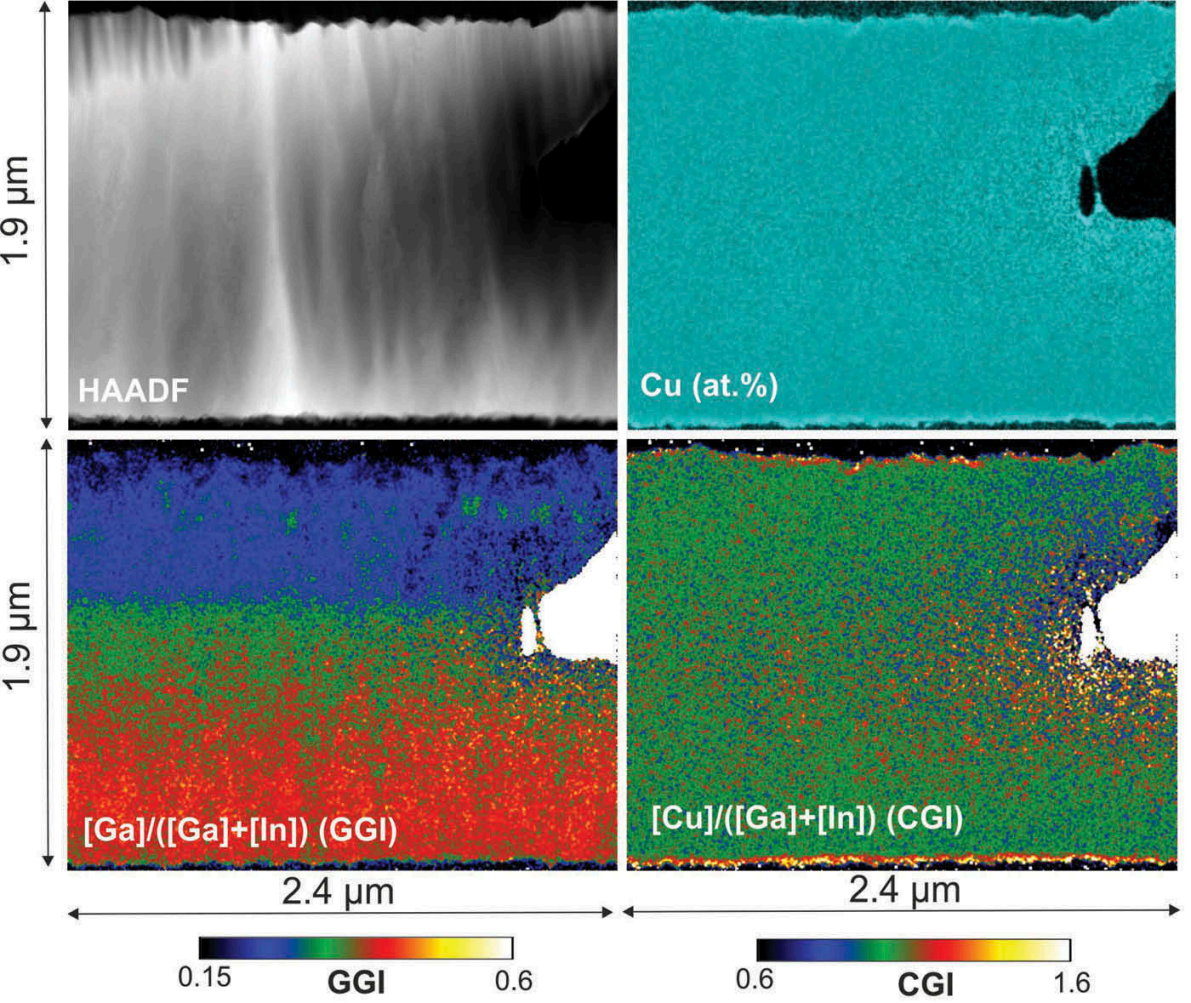
Sample 1: STEM-HAADF (top left), EDX Cu at.% map (top-right), GGI map (bottom-left) and CGI map (bottom-right).

A HAADF-STEM micrograph and STEM/EDX compositional mappings of Sample 1 are shown in Figure 2. Cu is homogeneously distributed across the specimen, except for the area surrounding of the hole at the right-hand side of the specimen, where some variations of the Cu concentration are due to the specimen preparation, which may result in Cu migration in the areas of the specimen closest to the unprotected edges. Also, CGI maps indicate a homogeneous Cu distribution in the lateral direction. GGI maps show the expected GGI grading across the thickness of the film up to the low-GGI region at the front (top of the image). The intentional GGI grading observed by STEM/EDX across the sample thickness was confirmed by SIMS depth profiling (not shown). This is consistent with the interruption of the deposition before the Cu-rich stage. The sample presents some randomly distributed GGI inhomogeneities. The lateral variations of the GGI values are 0.10-0.15. The inhomogeneous areas have granular shapes and diameters of 100–200 nm.

**Figure 3.**
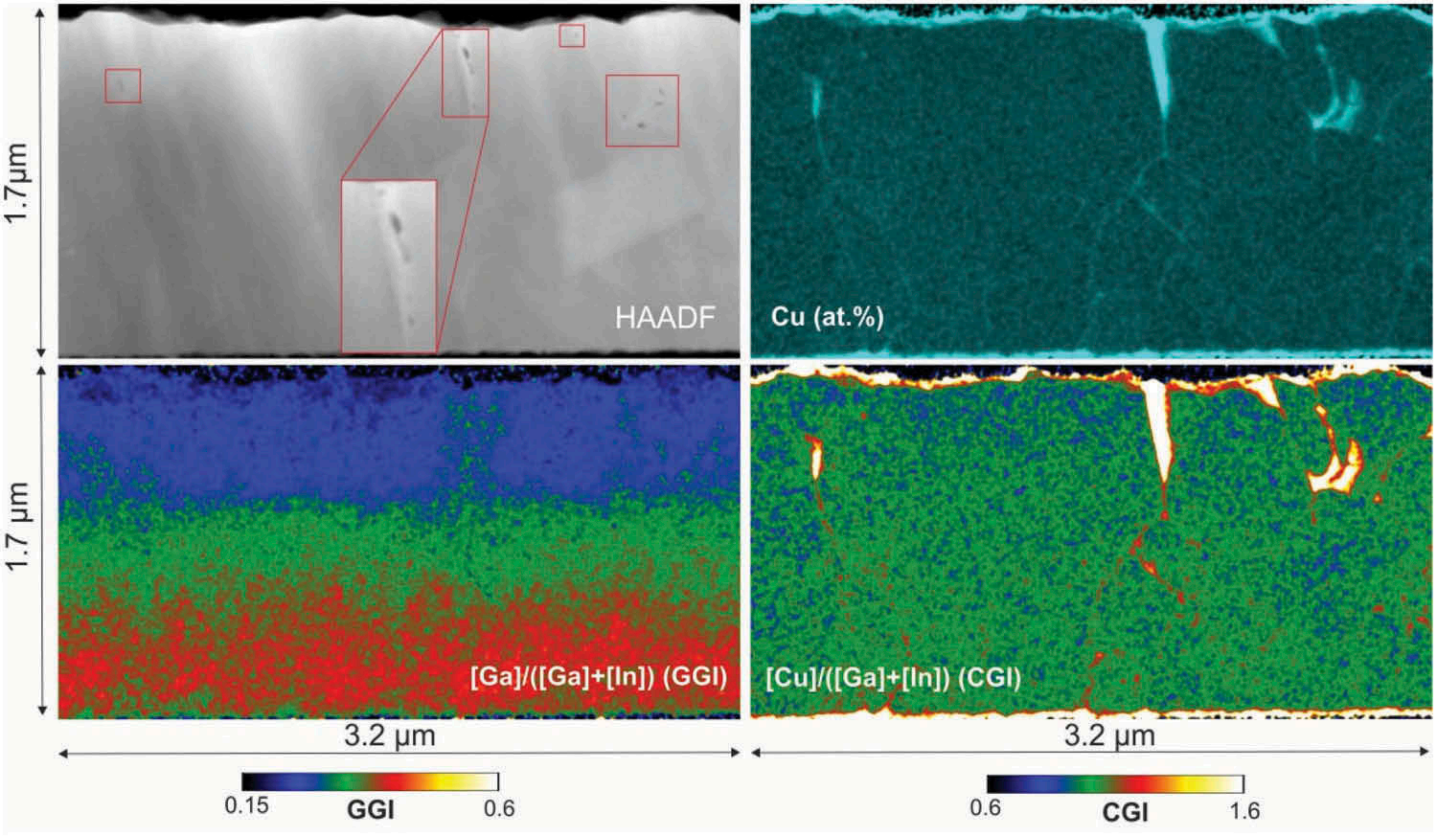
Sample 2: STEM-HAADF (top left), EDX Cu at.% map (top-right), GGI map (bottom-left), and CGI map (bottom-right).

Figure 3 shows HAADF-STEM micrographs and STEM/EDX compositional mapping of Sample 2. Cu-Se phases accumulate at grain boundaries and at crevices between CIGS grains, as shown by the Cu at.% and CGI maps. Very similar patterns have been observed in all other analyzed sections of the same specimen (Figure 3 supp.). The Cu-Se filled openings can reach sizes of up to several hundreds of nanometers. These regions are located in general, but not exclusively, at the surface of the Cu-rich CIGS film. In and Ga are not present in the crevice areas (supporting information Figure 4 supp.). The presence of small voids can also be observed in the HAADF-STEM micrograph in correspondence of Cu-Se segregation squares in Figure 3 (one void area has been enlarged for better view).

The GGI map of Sample 2 shows laterally uniform Ga to In ratios in the lower half of the film. In the upper half of the film some regions can be observed where the GGI values are laterally inhomogeneous, with widths of 100–200 nm and average GGI variations of approximately 0.05–0.10. Higher GGI values can be observed only in the regions surrounding those where Cu-Se segregation is present. All other regions show largely uniform lateral GGI distributions.

**Figure 4.**
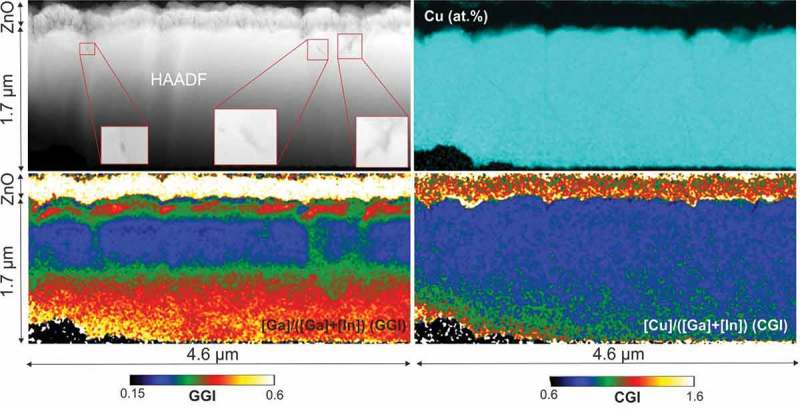
Sample 3: STEM-HAADF (top left), EDX Cu at.% map (top-right), GGI map (bottom-left), and CGI map (bottom-right).

Figure 4 shows HAADF-STEM micrographs and STEM/EDX compositional mapping of Sample 3 (finished device). The CGI and GGI calculated at the location of the ZnO film are not meaningful. In the CIGS film, the Cu intensity map shows some Cu-poor grain boundaries, elsewhere the Cu concentration is largely laterally homogeneous. The GGI presents instead large lateral inhomogeneities. The lower half of the film is fairly homogeneous laterally. The CGI variations at the bottom edge of the samples are believed to be caused by sample preparation, since this corresponds to an unprotected specimen edge, as already discussed for the results of Figure 2. Large columnar inhomogeneities are present in the central-top part of the film, with GGI variations of up to 0.10–0.15. The largest GGI inhomogeneities, with variations of up to 0.20, are observed in the top 200 nm close to the front surface. The GGI inhomogeneities are not correlated with grain boundaries but they are correlated with the presence of voids in the front regions of the film (in red squares in STEM-HAADF of Figure 4, three void areas have been enlarged for better view).

### 3.3. Voids

**Figure 5.**
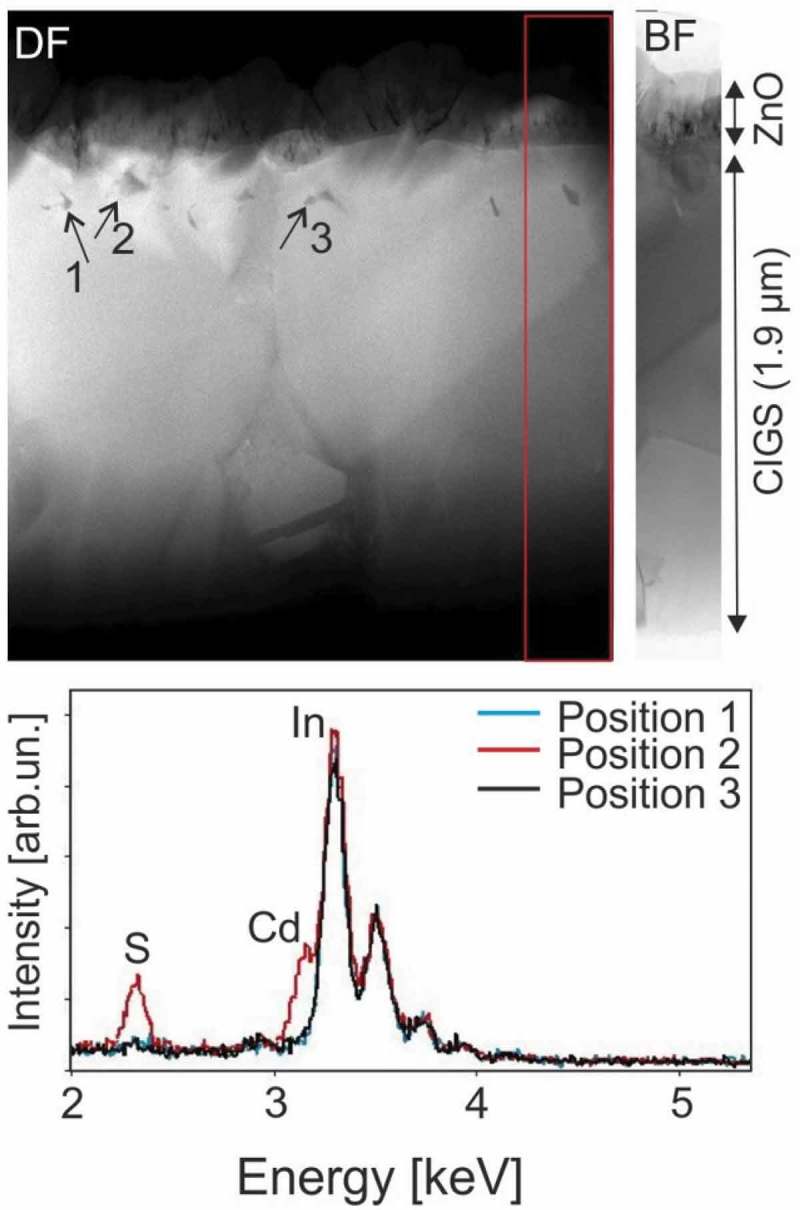
Sample 3. Top: HAADF-STEM micrograph of a completed CIGS solar cell and BF image of a selection of the same area (selection of BF image of the area at the right-hand side of the HAADF-STEM micrograph is shown for a better view). A large density of voids with diameters of up to 50 nm can be observed below the surface. Bottom: EDX spectra of selected areas.

**Figure 6.**
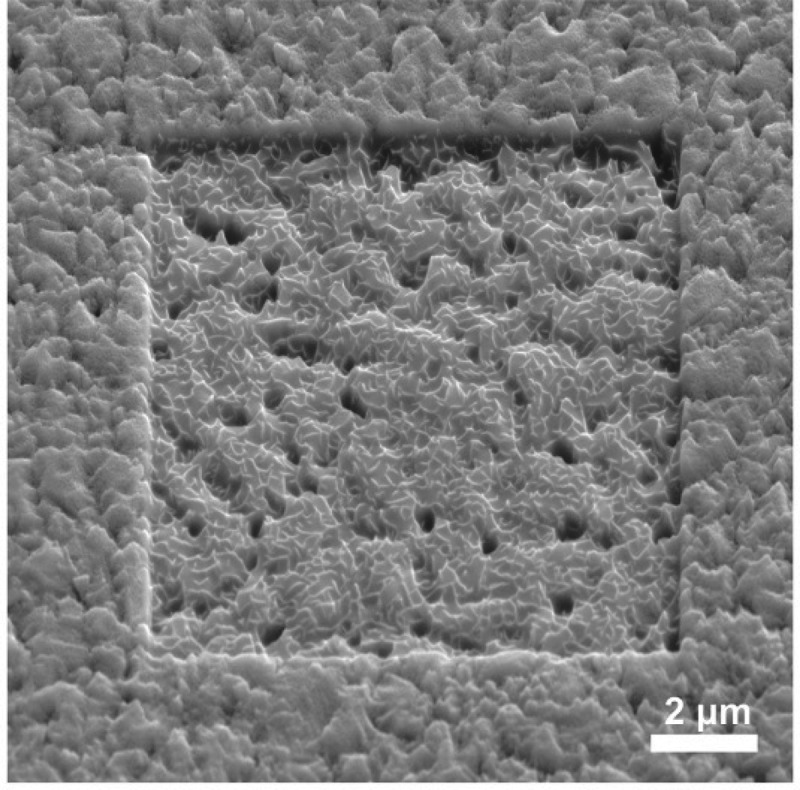
SEM micrograph of a completed CIGS sample surface (equivalent to Sample 3) after sputtering with Ga FIB on a 10 x 10 μm^2^ area (20 keV, 182 pA).

The presence of three macroscopic voids can be observed in the HAADF-STEM micrograph of Figure 4 (Sample 3, red squares) close to the surface. These voids are found close to the three areas with the largest GGI inhomogeneities. Smaller voids are also clearly visible in Sample 2 within the Cu-Se segregates (Figure 3). However, the HAADF-STEM resolution used for this analysis does not allow a precise assessment of the voids size and distribution. Therefore, another specimen from Sample 3 was analyzed with JEOL 2200FS TEM unit with an optimized HAADF-STEM and bright filed (BF) STEM signal. The results are shown in the Figures 5 and 6 supp.

Figure 5 shows a specimen area of Sample 3 where many voids are present. The specimen was prepared from a completed solar cell, which included the deposition of CdS by chemical bath deposition (CBD). Voids with diameters of 50–100 nm can be clearly observed. No specific shape could be identified. The voids are located 200–300 nm (± 100 nm) below the surface of the CIGS layers. This agrees well with the position of the voids observed in Figure 4. The specimen is a two-dimensional random section of the CIGS bulk which may or may not cross the voids present in the CIGS bulk. A very large number of specimens should therefore be analyzed in order to achieve a good statistics on the voids distribution and concentration. Since this is practically not feasible by TEM, an alternative approach was used, as described below.

In order to better assess the voids density and distribution, the surface of a CIGS sample was etched with a Ga-ion FIB and subsequently observed by SEM (Figure 6). A 10 x 10 µm^2^ area was etched for a depth of 300–400 nm. Approximately 80 voids can be counted, as they are exposed due to the etching of the top surface. The size of the voids is much larger than that measured by TEM as the Ga-ion beam preferentially etches the voids edges and enlarges their size. The density of the voids is approximately 0.5-1.0 µm^−2^, with a random lateral distribution.

STEM/EDX spectra were acquired along the borders of three voids (shown in Figure 5 as positions 1, 2, and 3). Matrix elements were identified in the areas surrounding all voids, as expected. EDX elemental mapping of the void at position 2 confirmed that peak intensities for matrix elements were either absent or much reduced within the void area (for example Figure 6 supp.). Along the voids at position 1 and 3, no additional species were identified by EDX. However, along the void at position 2, EDX signals of Cd (Lα1 at 3.1 eV) and S (Kα1, at 2.3 eV) were observed. The presence of Cd and S at the void’s internal surface indicates that the CBD solution could penetrate within at least some of the voids. Therefore, the voids at positions 1 and 3 are completely buried beneath the surface, whereas the void at position 2 is not.

## 4. Discussion

### 4.1. Formation of voids and compositional inhomogeneities

Small GGI inhomogeneities are observed before recrystallization (Sample 1) at randomly distributed positions across the sample. These inhomogeneities may be created during Cu addition, as Cu reacts with In and Ga selenides to form CIGS crystals. This could be a consequence of lateral Ga accumulation due to preferential In reactivity at facets of preferential growth. A similar mechanism has been used to explain the formation of Ga gradients in three-stage CIGS growth [12]. The appearance of small voids even before recrystallization could arise due to the formation of empty spaces between growing crystallites. However, no further proof for such mechanisms is given here and the understanding of these observations is to be taken as speculative.

The evolution of the elemental distribution from Sample 1 to Sample 2 suggests that the appearance of voids and crevices is a consequence of the recrystallization process during the Cu-poor to Cu-rich transition. Possibly, the crevices are formed by migration and accumulation of vacancies at Cu-rich conditions during re-crystallization, as suggested by Lei et al. [27]. Our results indicate that these areas are simultaneously or subsequently filled with Cu-Se phases segregating at surfaces and GBs.

In the sample with the completed growth, most voids are present starting from depths corresponding to the end of the Cu-rich deposition stage. This suggests that, after the Cu-rich stage, Cu diffuses from the crevices to the interior of adjacent grains. A void or an empty crevice is left behind. An analogous void formation mechanism was assumed by Schöldström et al. for the CURO process [28] (Cu-rich first stage followed by a Cu-poor stage) and by Kessler et al. [13] for the CUPRO process (similar to the three-stage used for our samples). Our findings provide evidence for the hypothesis of Kessler et al. [13] that Cu-Se phases segregate at crevices between GBs, and voids form by out-diffusion of Cu during the final deposition stage. It cannot be excluded that a partial conversion of Cu-Se phases to CIGS also occurs, as In and Ga may diffuse into the crevice area. As grain sizes strongly depend on the deposition temperature, the voids sizes and concentration might differ between low-temperature and high-temperature processes. Other process conditions such as the evaporation rates or the amount of Cu excess added after the first stoichiometry point may also influence void formation.

The small GGI inhomogeneities observed after the first stoichiometry point (Sample 2) seem to be present only in the regions surrounding the crevices. The In and Ga distributions are therefore homogenized by the re-crystallization process, except for the areas where Cu-Se phases accumulate. In the sample with a completed CIGS growth (Sample 3), very prominent GGI inhomogeneities are found in the near vicinity of voids and crevices on the CIGS surface (± 300 nm). No large voids or crevices are instead observed in the areas of laterally uniform GGIs. This suggests that the formation of voids may be correlated with the formation of compositional inhomogeneities and therefore with the presence of Cu-Se phases.

Indeed, segregated Cu_(2-x)_Se has been associated with an increased Ga and In inter-diffusivity [19,29]. Schroeder et al. [14] showed that when CuInSe_2_ is grown epitaxially with overstoichiometric Cu supply on a Ga-rich substrate, Ga diffuses from the substrate to the film, to the extent that the final CGI never exceeds 1. Consequently the diffusivities of Ga in CuInSe_2_ increased by two orders of magnitude from films grown stoichiometrically to films grown with an 0.2–0.4 Cu excess (from approximately 10^−13^ cm^2^/s to over 10^−11^ cm^2^/s, at temperatures above 700 °C) [14]. Therefore, diffusion of group-III elements may be enhanced both within the Cu-Se phases, and in the CIGS neighboring those phases as a way to compensate the over-stoichiometric Cu supply. A consequence is the widely reported smoothening of the GGI grading occurring when a larger Cu excess is provided at the intermediate stage of the three stage coevaporation growth [29–32].

Similarly, Cu-dependent group-III diffusivities can explain GGI inhomogeneities in Sample 2 and Sample 3. The inhomogeneous GGI regions surrounding crevices in Sample 2 have in fact a larger Ga concentration. This indicates enhanced diffusion of Ga from the Ga-rich back regions of the film. During the final deposition stage, Cu-Se phase segregation is still expected until it is completely phased out. The large GGI inhomogeneities in the completed CIGS film are formed during the final In and Ga evaporation, and can be caused by increased In and Ga diffusivities in the vicinity of the crevices filled by Cu-Se phases. This is supported by the correlation between voids (or surface crevices) and GGI inhomogeneities in Sample 3. The front of the completed absorber is deposited during the transition from Cu-rich to stoichiometric and finally Cu-poor (3^rd^ stage), that is, consumption of the Cu-Se phases at the sample surface. This transition corresponds to an abrupt change in Ga and In diffusivities in a narrow CGI range [14]. The largest GGI inhomogeneities at the very front of Sample 3 are likely the consequence of an inhomogeneous distribution of the second stoichiometry point during the final deposition stage. These observations are supported by a larger statistics of analogous observations in several other sections of the specimens of Sample 2 and Sample 3 (supporting information Figure 3 supp. and Figure 5 supp.).

### 4.2. Scale of the inhomogeneities

**Figure 7.**
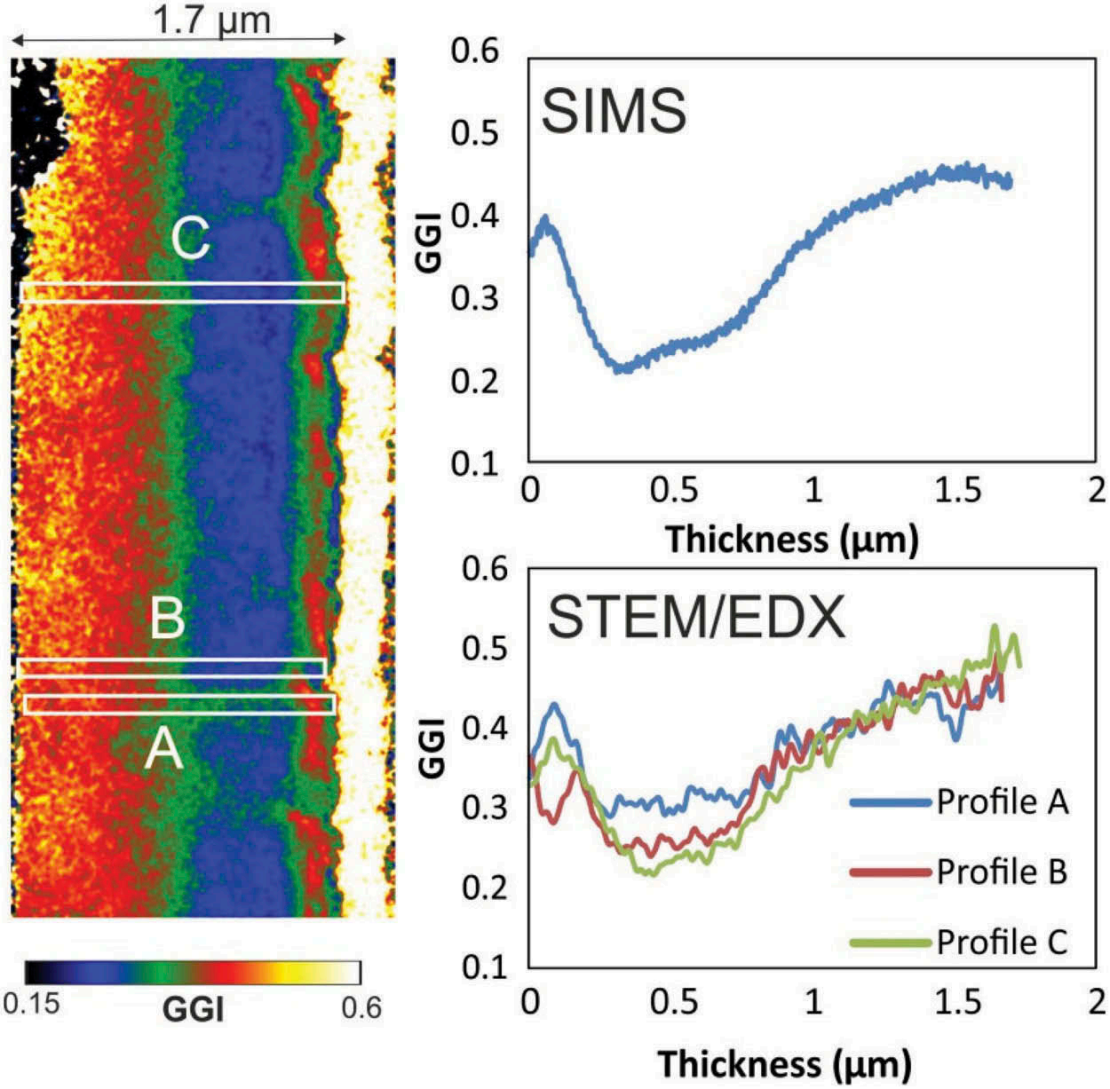
Sample 3: Left: STEM/EDX GGI map. Top-right: GGI grading as measured by SIMS depth profiling. Bottom-right: GGI gradings from the STEM/EDX GGI map along cross-sectional lines with 20 nm thickness (the value at each point is the average over the given thickness). Thickness scaling of SIMS depth profile is made according to the one measured by STEM for comparison.

Lateral variations of the GGI grading were estimated by measuring the GGI grading along lines across the depth of Sample 3 (average values over 20-nm-thick lines). Figure 7 shows that the GGI grading is fairly homogeneous at the back of the film, except for minor fluctuations. In the central-front region of the film, large differences in the extracted GGI profiles are present, with variations of up to 0.1. At the very front, the GGI grading differences are the largest, with variations of up to 0.15. The GGI grading measured by SIMS over a wider area agrees well with intermediate values (100 × 100 µm^2^, GGI profile calibrated using integral value measured by XRF).

GGI values were taken also along lines parallel to the surface (average values over 20-nm-thick lines). The standard deviations of the GGI along those lines are largest in areas close to the front surface, with values of up to 0.034 close to the CIGS/CdS interface. This would correspond to a standard deviation of the lateral bandgap values at the surface of 24 meV.

### 4.3. Consequences for solar cell performance

**Figure 8.**
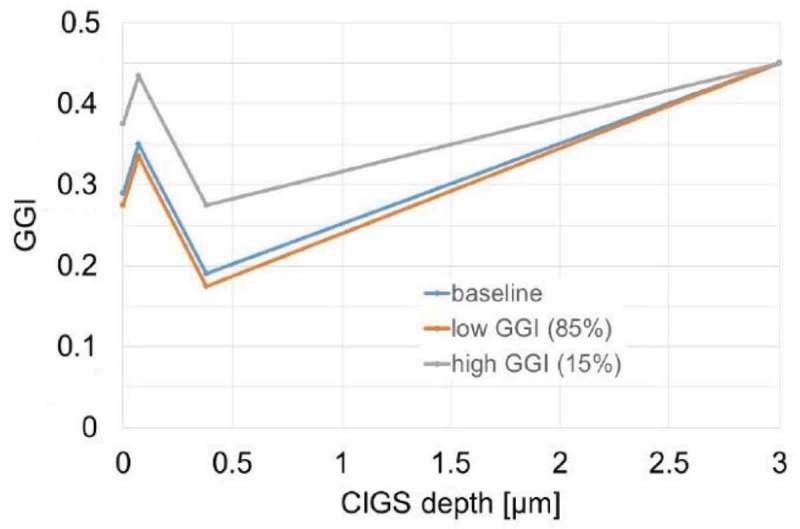
GGI gradings used for Sentaurus 2D simulations. ‘High-GGI’ profile and ‘low-GGI’ profile have been employed in a inhomogeneous 2-section structure with a 15%–85% width ratios, respectively. The baseline grading corresponds to the average composition at each depth, weighted over the respective width. The baseline grading profile was also used to simulate the performance of a reference homogeneous structure.

We performed two-dimensional device simulations to estimate the impact of the inhomogeneities on the performance of CIGS solar cells (Software: Sentaurus TCAD Suite). More details on the two-dimensional simulations can be found in an earlier publication [33]. Structures with 100 nm width were simulated, comprising a double-graded CIGS absorber divided into two areas with a low-bandgap and a higher-bandgap grading, with widths of 85 nm and 15 nm, respectively. The GGI variations were most pronounced in the central-front regions of the absorber, as observed in the actual samples. The simulated gradings are shown in Figure 8.

Several simulations were performed assuming interface acceptor trap densities ranging from 2·10^13^ cm^−3^ to 8·10^18^ cm^−3^, donor trap densities from 10^15^ cm^−3^ to 10^20^ cm^−3^, capture cross sections for electrons and holes at the interface from 10^−14^ to 10^−9^ cm^2^ and donor trap densities in the bulk from 5·10^15^ cm^−3^ to 10^16^ cm^−3^. At the structures’ lateral boundaries, total electron and hole reflection were assumed. At the absorber/buffer junction, the CdS conduction band was kept at constant energy difference from the Fermi level, so that a change in the CIGS bandgap (GGI) at the interface would lead to a different interface conduction band offset (CBO). The simulations were compared to simulated uniform structures with a baseline grading. The baseline grading corresponded to the average between the gradings of the inhomogeneous sections, weighted over the corresponding widths.

In none of the cases the GGI inhomogeneities led to considerable differences in the simulated device performance, as compared to the uniform structures. The maximum simulated V_oc_ difference is 3 mV between the homogeneous and the inhomogeneous case. The maximum efficiency loss is 0.2%. Therefore, our simulations indicate no major impact of the inhomogeneous GGI grading and interface CBO on the optical properties or on the interface quality of real devices in the GGI range investigated. This is consistent with the analysis by Abou-Ras et al. [3], who suggested a negligible impact of the compositional inhomogeneities on the V_oc_. An upper limit to the impact of the inhomogeneities on the V_oc_ can be found also by using an earlier model [5,34]. According to this model, Abou-Ras et al. [3] suggested an expression for the dependency of the V_oc_ on the lateral bandgap standard deviation (equation 5 in reference [3]). In our case, a standard deviation of approximately 24 meV was calculated close to the CdS/CIGS junction, which would lead to a V_oc_ loss limited to maximum 10 mV.

An additional enhancement of the bandgap fluctuations may be induced by lattice strain induced by GGI-dependent variations of the tetragonal distortion. Even for relatively small GGI variations (0.05–0.1) [34], these may lead to an enhancement of the bandgap fluctuations of up to 60 meV [34], which would affect the V_oc_ by several tens of mV. However, precise data on the strain are not available and a direct effect on the V_oc_ can therefore not be calculated. In addition, our simulations do not take into account a possible increase in the local deep defect density at higher-GGI interface regions, which has been reported at the CdS/CIGS interface [35]. The inhomogeneous grading may therefore lead to localized channels of high interface recombination velocities. In absence of precise values for interface and bulk defect densities, a simulation of inhomogeneous bandgap structure would also be unreliable.

We conclude that the GGI inhomogeneities, in the investigated range, are not a source of performance loss if only the direct dependency of the GGI on the bandgap is considered. However, possible losses may arise due to the additional effects of lateral strain on the bandgap fluctuations, and due to varying deep defect densities. With the current state of knowledge, the consequences of local lateral strain and varying deep defect densities on the solar cell performance cannot be reliably predicted.

**Figure 9.**
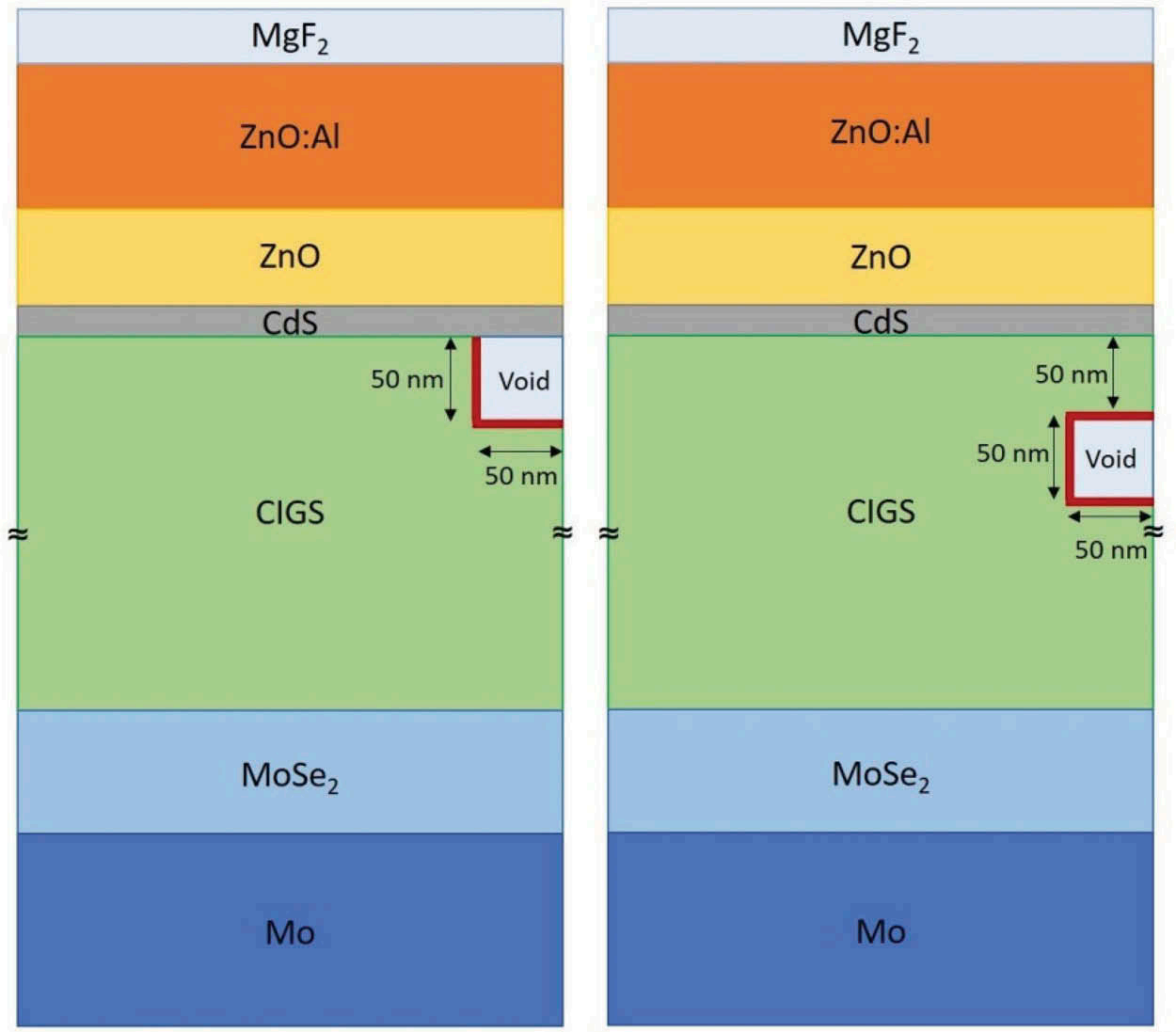
Structures employed in three-dimensional void simulations. Three-dimensionality is obtained by rotation around the edge at the right-hand side of each structure, creating cylindrical shapes for both the solar cell stacks and the voids. Right: the void is adjacent to the CdS buffer layer. Left: the void is buried 50 nm beneath the CIGS surface.

The impact of voids was investigated by three dimensional simulations using the Sentaurus TCAD Suite software. Cylindrical structures with a diameter of 1 μm were employed. The following three scenarios are simulated: no voids; one cylindrical void adjacent to the CdS; one cylindrical void buried 50 nm beneath the CIGS surface. In both cases the simulated voids had a diameter of 100 nm and a height of 50 nm. The structures are shown in Figure 9. Illuminated J-V characteristics (AM1.5G) were calculated using input parameters shown in the supporting information (Table 1 supp.). The employed optical properties were previously measured on CIGS samples with different compositions and on all additional layers by transmittance-reflectance spectroscopy and spectroscopic ellipsometry (details published elsewhere [22]). At the voids’ internal surfaces, surface recombination velocities were varied between 2 · 10^3^ cm/s and 1 · 10^5^ cm/s.

Analysis of time-resolved photoluminescence (TRPL) measurements [36] indicate that surface recombination velocities have values between 10^3^ cm/s and 10^4^ cm/s at free CuInSe_2_ (CIS) surfaces. Neither the TRPL model nor the 3D Sentaurus simulations assume surface band bending or trapped charge densities at free surfaces. These have to be taken therefore as effective surface recombination velocities which lead to correct surface recombination rates. No reliable numbers are yet available for CIGS, but surface recombination velocities may be higher in Ga-containing material.

The results of 3D simulations are shown in Table 1.

**Table 1.** Simulated J-V parameters of different cylindrical structure with and without voids, depending on surface recombination velocities.

	Surf. rec. velocity (cm/s)	V_oc_ (V)	FF (%)	Δη (% abs.)
Baseline (no voids)		0.742	80.6	Ref.
Void (interface)	2 · 10^3^	0.732	79.9	−0.5
	4 · 10^3^	0.724	79.4	−0.8
	1 · 10^4^	0.709	78.4	−1.6
	1 · 10^5^	0.648	74.6	−4.3
Void (buried)	2 · 10^3^	0.733	80.0	−0.4
	4 · 10^3^	0.726	79.6	−0.7
	1 · 10^4^	0.711	78.7	−1.4
	1 · 10^5^	0.652	75.2	−4.0

For surface recombination velocities values between 2 · 10^3^ cm/s and 10^4^ cm/s, the void-related losses vary from limited (9 mV V_oc_, 0.6% FF, and 0.4% η) to severe (more than 30 mV V_oc_, 2% FF, and 1.6% η). The J_sc_ is largely unchanged in this range of surface recombination velocities (maximal reduction 0.2 mA/cm^2^), and hence neglected. The effects on the FF and V_oc_ are slightly more pronounced if the void is adjacent to the CdS/CIGS interface, rather than buried beneath the surface. It must be noted, however, that a void at the CIGS/CdS interface would likely have a CdS-passivated surface. The CdS-CBD solution would in fact reach the inner void internal area, as shown in Figure 5. Results for a larger range of surface recombination velocities are presented in the supporting information (Figure 7 supp.).

The structure diameter and void sizes were chosen following the measured statistical distribution and size (Figures 5 and 6). However, the possibility of partial passivation of the voids’ internal surface was omitted in the simulations, due to the lack of reliable statistics. CdS coverage would passivate the void surface by establishing an inversion layer at the CIGS side and strongly reduce the surface recombination velocities (an upper limit of 1.4 · 10^3^ cm/s was evinced from TRPL measurements [36]). Simulated passivated voids, with zero recombination velocities on the sidewalls, have no effect on the cell’s performance.

It can be concluded, that voids introduce free surfaces close to the CdS/CIGS interface which may severely impact the electronic performance of solar cells. Efficiency limitations of above 1% absolute are expected for surface recombination velocities above 5 · 10^3^ cm/s. These effects may be possibly mitigated by the use of wet buffer layer deposition methods: some of the voids’ internal surfaces can be reached by the deposition solution and are therefore be partially passivated, as previously observed by Lei et al. [37]. Statistics in this sense is still missing. It may still be speculated, that a partial void surface passivation could explain the typically observed superiority [1] of wet buffer layer deposition methods as compared to dry ones in case of multi-stage co-evaporated CIGS.

## 5. Conclusions

We analyzed two types of structural defects potentially affecting the performance of CIGS solar cells with absorbers layers grown by three- or multistage coevaporation processes. Lateral GGI inhomogeneities were observed in the front region of CIGS thin films grown by multistage coevaporation at low temperature. Lateral GGI variations up to 0.10–0.15 are observed in the central-front of the absorber and up to 0.20 at the front surface. Voids were observed beneath the CIGS surface with diameters of up to 100 nm and a lateral occurrence of half to one void per square micrometer. To understand the formation mechanism of such defects, CIGS growths were interrupted at different stages of the co-evaporation process. It was concluded that the largest GGI inhomogeneities are formed as a consequence of inhomogeneous Cu distributions after the Cu-rich deposition stage. Voids are formed due to out-diffusion of Cu from Cu-Se segregation at crevices in the film during the final deposition stage. The voids’ size and distribution might be influenced by different process parameters. Three-dimensional simulations indicate that voids lead to degraded solar cell performance with an efficiency loss of approximately 1% for surface recombination velocities consistent with those estimated by TRPL measurements on CIS surfaces, which may be higher for CIGS. Further investigations are still needed in this sense. The deposition of buffer layers by wet chemistry methods may lead to a partial passivation of the voids internal surfaces, as the CBD solution can penetrate within the voids, which may mitigate their detrimental effect. Although void formation is likely unavoidable in a three-stage process, we suggest that further process modifications should be investigated in order to increase the exposure of the voids to the surface and thus the relative density of voids passivated by the buffer layer.
