# Supplementary material for: Voids and compositional inhomogeneities in Cu(In,Ga)Se2 thin films: evolution during growth and impact on solar cell performance
Source: Sci Technol Adv Mater. 2018 Nov 19;19(1):871–82. doi: 10.1080/14686996.2018.1536679 (PMC6249540; doi:10.1080/14686996.2018.1536679)
Supplement: Supplemental Material [file TSTA_A_1536679_SM7424.pdf]

# Voids and compositional inhomogeneities in $Cu(In,Ga)Se_2$ thin films: evolution during growth and impact on solar cell performance

Enrico Avancini[1], Debora Keller[2], Romain Carron[1], Yadira Arroyo[2], Rolf Erni[2], Agnieszka Priebe[3], Simone di Napoli[4], Martina Carrisi[4], Giovanna Sozzi[4], Roberto Menozzi[4], Fan Fu[1], Stephan Buecheler[1], and Ayodhya N. Tiwari[1]

[1] Thin Films and Photovoltaics, Empa-Swiss Federal Laboratories for Materials Science and Technology, Ueberlandstrasse 129, CH-8600 Duebendorf, Switzerland

[2]Electron Microscopy Center, Empa-Swiss Federal Laboratories for Materials Science and Technology, Ueberlandstrasse 129, CH-8600 Duebendorf, Switzerland

[3]Empa, Swiss Federal Laboratories for Materials Science and Technology, Laboratory for Mechanics of Materials and Nanostructures, Feuerwerkerstrasse 39, CH-3602 Thun, Switzerland

[4]Department of Engineering and Architecture, University of Parma, Parco Area delle Scienze 181A, 43124 Parma, Italy

## Supporting information

### 1. Alkali metal diffusion from soda-lime glass substrate

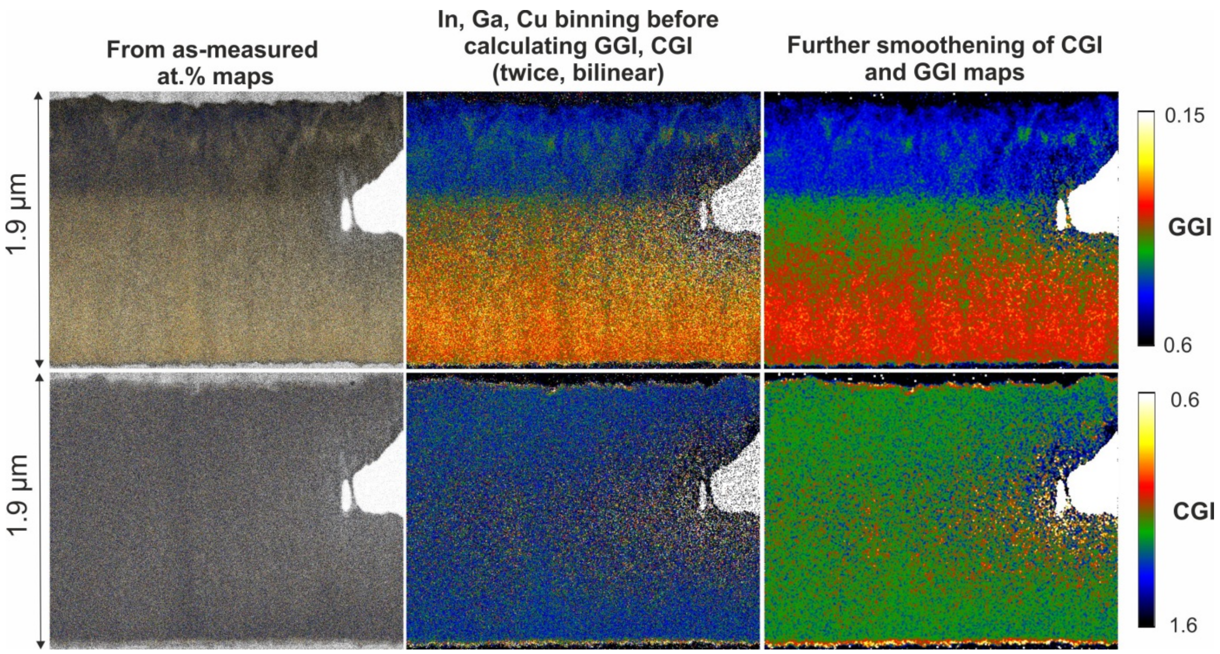


Figure 1 (sup.). Effects of binning and smoothening data processing by the DigitalMicrograph software on Sample 1. In the non-binned and non-smoothened maps, the SNR is far too large to interpret features in the GGI and CGI distributions. A more intelligible image is obtained after two-fold binning and smoothening.

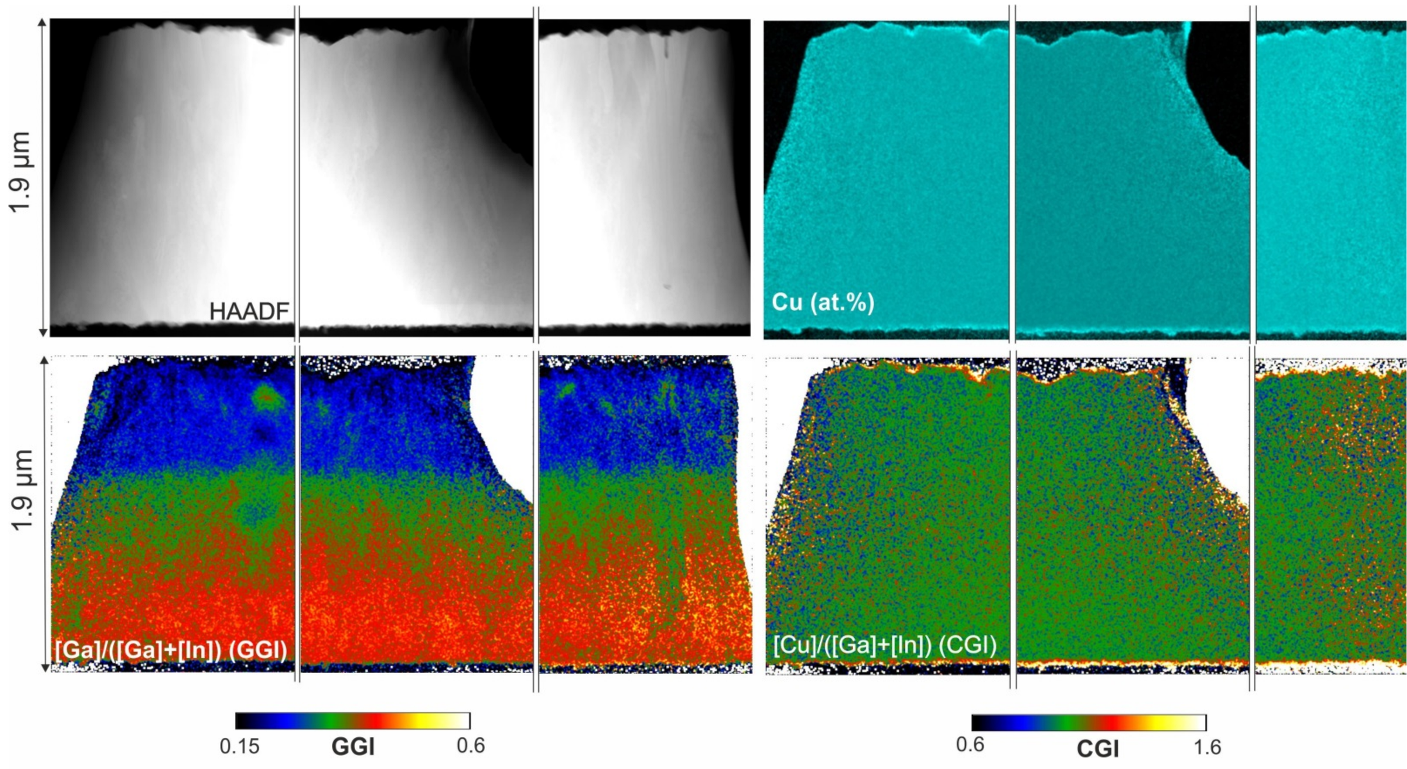


**Figure 2 (sup.). Additional sections of Sample 1 (non-recrystallized CIGS film): STEM-HAADF, EDX at.% map, GGI, CGI maps to be compared and supporting with the findings already presented in Figure 3.**

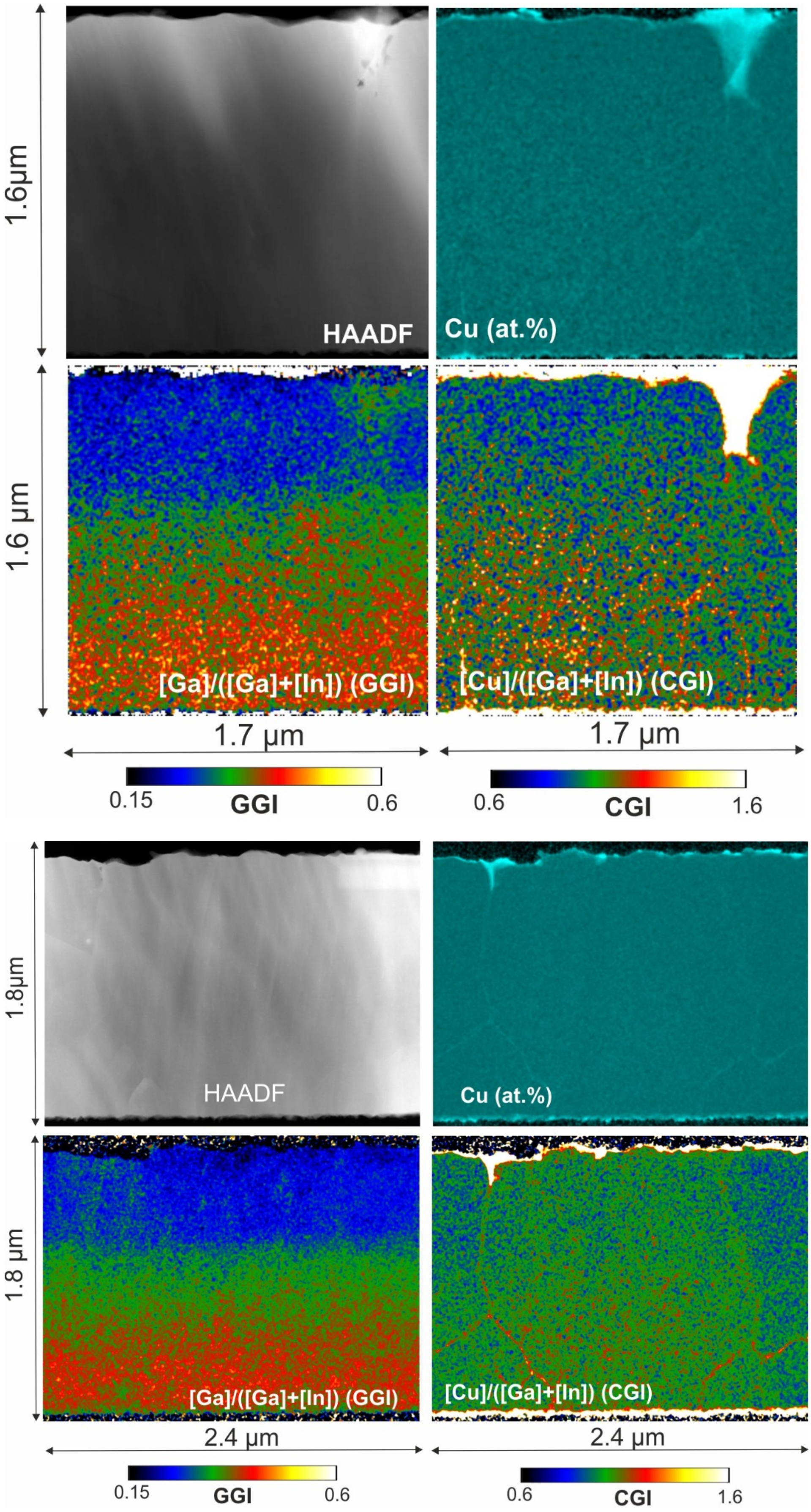


**Figure 3 (sup.). Additional sections of Sample 2 (Cu-rich, re-crystallized CIGS film): STEM-HAADF, Cu at.% maps, GGI, CGI maps supporting the findings shown in Figure 3. This supports the correlation between Cu-Se phase segregation and increased Ga**

**concentration. The GGI and CGI maps of the lower figure were calculated starting from 2-times binned Cu, In and Ga at.% maps and further smoothening was applied, as specified in the experimental section.**

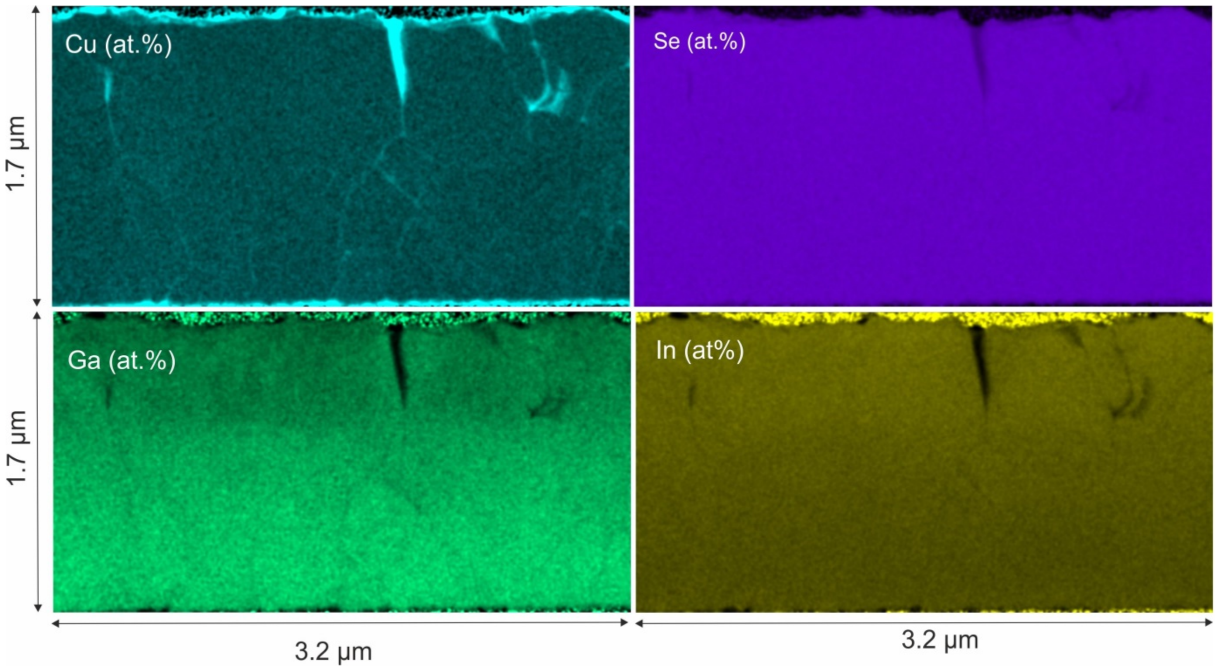


**Figure 4 (sup.). Additional at.% maps from Sample 2 (Cu-rich, re-crystallized CIGS film), showing that matrix elements In and Ga are missing from the void area. This confirms that the phases segregated at the void area are mainly composed of Cu and Se.**

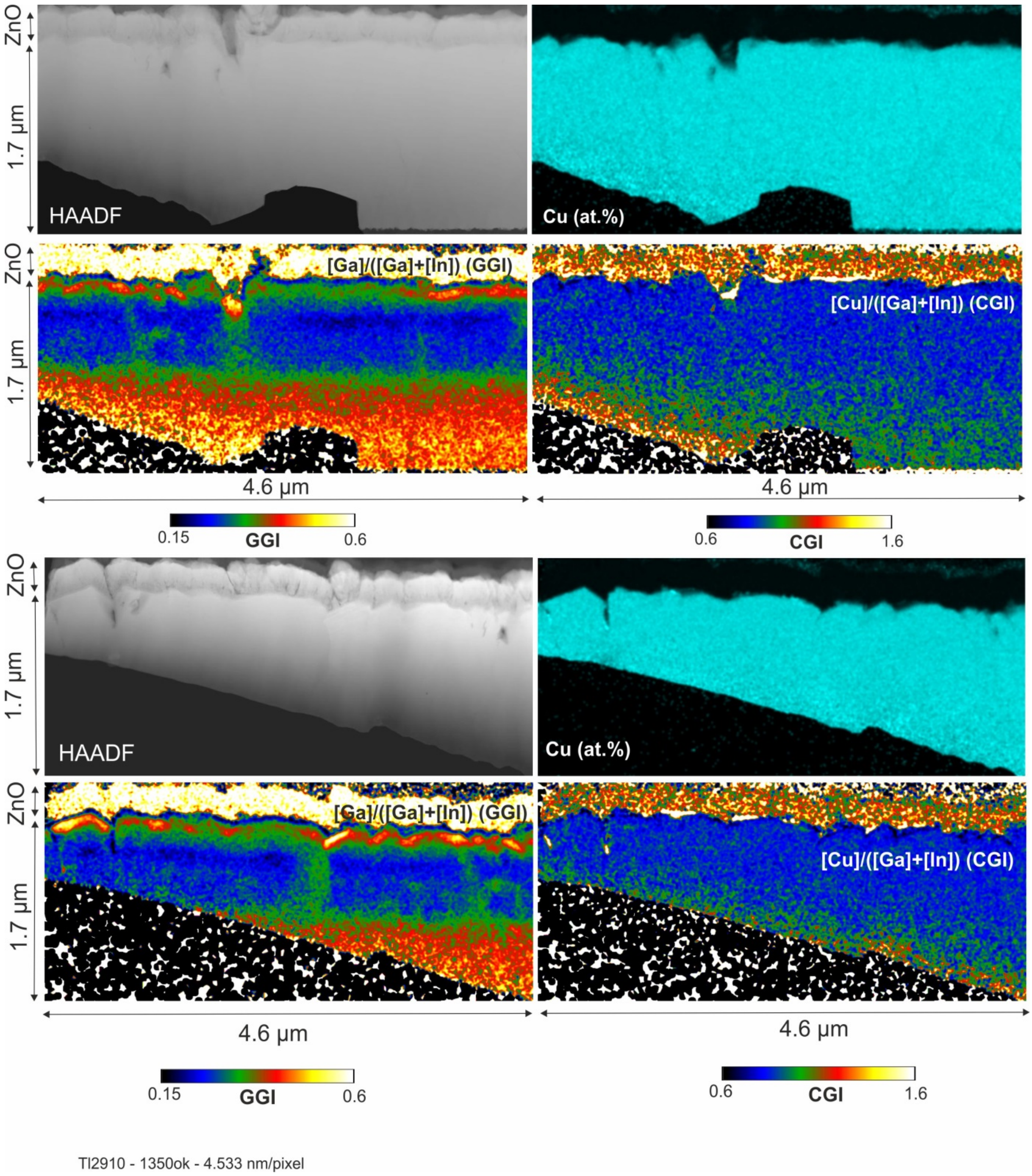


**Figure 5 (sup.). Additional sections of Sample 3 (completed CIGS film with the addition of CdS and ZnO layers): HAADF, Cu at.% map, GGI, CGI maps. The lower section does not correspond to the full sample thickness as part of the layer broke during sample preparation. The non-uniformities observed in Figure 4 are also at these additional regions of Sample 3. The correlation between the presence of voids and crevices and the GGI non-uniformities is also supported.**

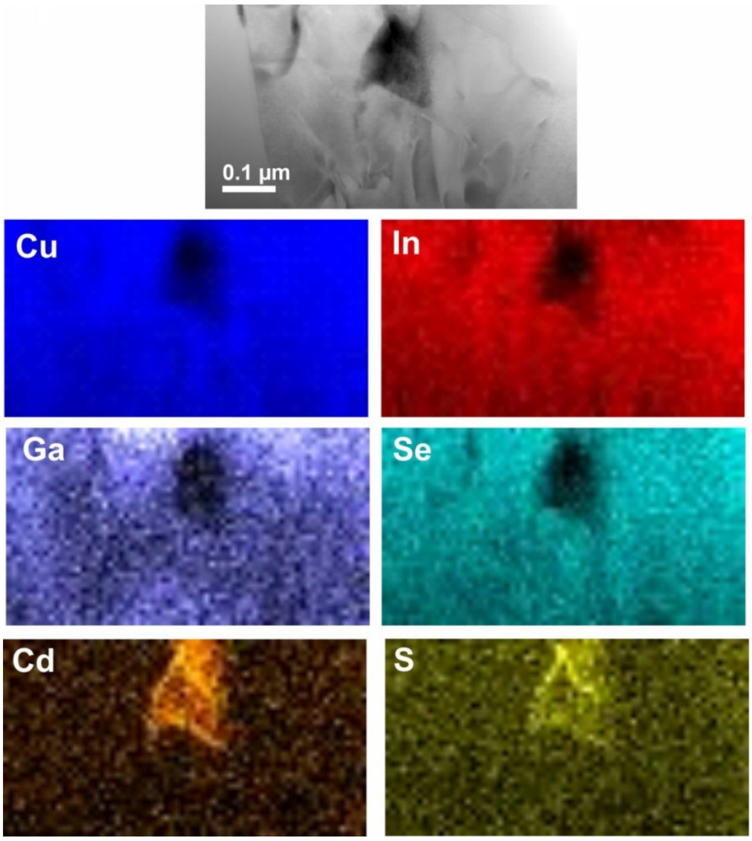


Figure 6 (sup.). Elemental (EDX intensity) maps from a void area of Sample 3, showing that matrix elements In, Ga, Cu and Se are below detection limit within the void area, filled with Cd and S instead. This indicates an opening of the void towards the surface where S and Cd can penetrate during the CdS-CBD.

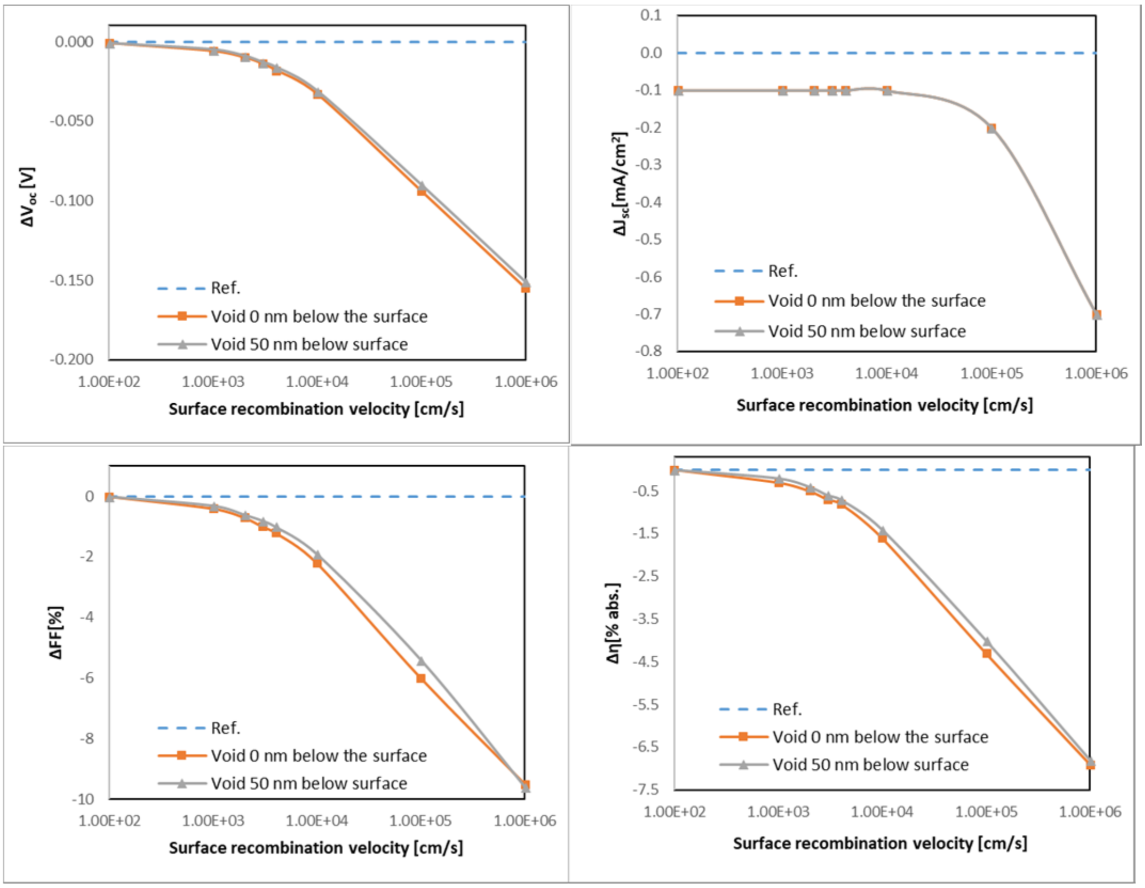


**Figure 7 (supp.). Simulated variations of solar cell parameters depending on the position of the void (as described in the discussion section of the manuscript) and on the surface recombination velocity.**

**Table 1 (supp.). Parameters employed in the three-dimensional simulations of cylindrical cell structures, with and without the voids. Details on optical coefficients are published elsewhere[22].**

| Material | $E_g$ [eV] | $\chi$ [eV] | Thickness [µm] | Doping [$cm^{-3}$] | Bulk traps [$cm^{-3}$] | σe , σh [$cm^2$] | Optical coeffcients |
|---|---|---|---|---|---|---|---|
| ZnO:Al | 3.3 | 4.5 | 0.2 | $4\cdot10^{19}$ | Acceptor ($1\cdot10^{16}$) | $1\cdot10^{-15}$<br>$1\cdot10^{-12}$ | Empa measured |
| ZnO | 3.3 | 4.5 | 0.08 | $1\cdot10^{17}$ | Acceptor ($1\cdot10^{16}$) | $1\cdot10^{-15}$<br>$1\cdot10^{-12}$ | Empa measured |
| CdS | 2.4 | 4.3 | 0.03 | $1\cdot10^{17}$ | Acceptor ($3\cdot10^{15}$) | $1\cdot10^{-15}$<br>$1\cdot10^{-12}$ | Empa measured |
| CIGS | Graded | 4.6 | 3 | $1\cdot10^{16}$ | Donor ($6.67\cdot10^{14}$) | $1\cdot10^{-15}$<br>$1\cdot10^{-15}$ | Empa measured (CGI=0.78) |
